# Hookworm Due to Surface Privies and Lack of Privies

**Published:** 1909-12

**Authors:** 


### Hookworm Due to Surface Privies and Lack of Privies.

Ch. Wardell Stiles, Ph. D., Chief of the Division of Zoology, Hygienic Laboratory, United States Public Health and Marine-Hospital Service, says: Recent investigations on the subject of hookworm disease in the South have brought to light certain conditions of soil pollution which call for serious consideration because of their influence on the spread of disease, especially hookworm disease and typhoid fever.

By actual count of 366 farmhouses scattered over four Southern States it was found that only 115 houses, or 31.4 per cent, were provided with privies, while 251 houses, or 68.5 per cent, had no privy. Thus, a condition of theoretical maximum soil pollution was occurring around 68.5 per cent of the houses in question. When it is considered that both hookworm disease and typhoid fever are spread through night soil, the importance of this soil pollution becomes evident.

Even when a privy is present soil pollution may occur in case the outhouse is not properly built or not properly cleaned.

Among several thousand privies examined, on farms and in various villages, the prevailing style was found to be the surface privy open in back. This is the poorest compromise that can be made, for not only is the danger present of contaminating the water supply in near-by wells, but soil pollution naturally occurs around the outhouse, and this is increased by the fact that chickens, dogs and hogs have access to the night soil and scatter the infectious material around. Further, also, flies and other insects either breed in or feed upon the excreta and carry fecal material to the food in the houses. An urgent necessity is present for a radical reform in this matter and for the abolition of this style of privy.—*Southern California Practitioner.*

---

To develop manual dexterity nothing is better than the practice of tying surgical knots.—*American Journal of Surgery.*

